# PERSPECTIVES OF SPEECH LANGUAGE PATHOLOGISTS AND STUDENTS ON PROVIDING CARE TO PEOPLE WITH DEMENTIA

**DOI:** 10.1093/geroni/igad104.2606

**Published:** 2023-12-21

**Authors:** Makenna Green, Jamie Mayer, Laura White

**Affiliations:** Northern Illinois University, DeKalb, Illinois, United States; Northern Illinois University, DeKalb, Illinois, United States; University of South Alabama, Mobile, Alabama, United States

## Abstract

This scoping review aimed to explore the extant literature on the experiences and views of speech-language pathologists (SLPs) and SLP students regarding the provision of care to people living with dementia (PLWD). A systematic search was conducted using 10 databases. Sources were included if participants were practicing SLPs and/or students enrolled in undergraduate communicative disorders or graduate SLP programs, and if the concepts of experiences or views on the provision of SLP services to PLWD were explored in the context of any clinical or educational setting. Included sources were systematically extracted for pertinent study characteristics, including SLP roles and settings, concept domains, measures utilized, and facilitators/barriers to effective dementia care. The majority of the 29 included sources were published in either academic journals (n=20) or professional organization publications (n=5) and used a cross-sectional study design (n=19). Participants included SLPs (n=22), and masters (n=6), undergraduate (n=3 studies), and doctoral students (n=1). The included studies addressed five primary conceptual domains: experiences, attitudes, roles, knowledge, and confidence. The most commonly addressed barriers and facilitators of effective dementia care were education and training. Mapping and analysis of the current body of knowledge within this scoping review illuminated several knowledge gaps that we propose need to be addressed to meet the education and training needs of SLPs to provide optimal care to PLWD. These include addressing core competencies, systematically measuring educational intervention outcomes, and developing more evidence-based education and training programs both within and outside of an interprofessional collaborative context.

